# A Comparison of Casual In-Clinic Blood Pressure Measurements to Standardized Guideline-Concordant Measurements in Severely Obese Individuals

**DOI:** 10.1155/2015/801709

**Published:** 2015-07-29

**Authors:** Sana Vahidy, Sumit R. Majumdar, Raj S. Padwal

**Affiliations:** ^1^Department of Medicine, University of Alberta, Edmonton, AB, Canada T6G 2G3; ^2^Alberta Diabetes Institute, Edmonton, AB, Canada T6G 2E1

## Abstract

*Background/Objectives*. The objective of this study was to compare casual BP taken in a bariatric clinic to standardized guideline-concordant BP. *Subjects/Methods*. A cross sectional analysis was performed using baseline data from a weight management trial. Patients were recruited from a Canadian bariatric care program. Standardized BP was performed using a Watch BP oscillometric device. Casual in-clinic BP single readings, taken using a Welch Allyn oscillometric device, were chart-abstracted. Paired *t*-tests, Bland-Altman plots, and Pearson's correlations were used for analysis. *Results*. Data from 134 patients were analyzed. Mean age was 41.5 ± 8.9 y, mean BMI was 46.8 ± 6.5 kg/m^2^, and 40 (30%) had prior hypertension. Mean casual in-clinic BP was 128.8 ± 14.1/81.6 ± 9.9 mmHg and mean standardized BP was 133.2 ± 15.0/82.0 ± 10.3 mmHg (difference of −4.3 ± 12.0 for systolic (*p* < 0.0001) and −0.4 ± 10.0 mmHg for diastolic BP (*p* = 0.6)). Pearson's coefficients were 0.66 (*p* < 0.0001) for SBP and 0.50 (*p* < 0.0001) for DBP. 28.4% of casual versus 26.9% of standardized measurements were ≥140/90 mmHg (*p* < 0.0001). *Conclusion*. In this bariatric clinic, casual BP was unexpectedly lower than standardized BP. This could potentially lead to the underdiagnosis of hypertension.

## 1. Introduction

Severe obesity, which is defined as a body mass index (BMI) ≥ 40 kg/m^2^, has quadrupled in prevalence over the past two decades and is the fastest growing obesity subgroup, now affecting over 3% of adults in Canada and over 6% of adult Americans [1–3]. Severely obese individuals are at risk for substantial multimorbidity and premature mortality and have double the health care costs of individuals with normal BMI levels [4–6].

Hypertension is a very common comorbidity in the severely obese, affecting 65% of these individuals [7, 8]. One major challenge in severely obese patients with or at risk for hypertension is obtaining accurate blood pressure readings [9, 10]. Major factors contributing to inaccurate measurement, and generally leading to falsely elevated readings, include very large arm circumferences (leading to undercuffing when inappropriately small cuffs are used), short arm length in proportion to arm size (causing the cuff to extend past the antecubital fossa), and conically shaped arms (resulting in nonuniform compression of the artery when standard, cylindrical cuffs are used) [10, 11].

In addition to these pitfalls, blood pressure measurement in clinical settings is frequently also inaccurate because care providers do not follow standardized measurement recommendations [11–14]. Common additional errors include taking single readings, talking while performing measurements, failing to allow a sufficient rest period prior to measurement, improper patient positioning, and terminal digit preference [11, 12]. Most of these factors can also spuriously raise blood pressure, as can the white coat effect, which is found in 15–45% of clinic patients [15, 16]. Overdiagnosis of hypertension and overtreatment with antihypertensive drugs are the expected consequence of falsely elevated readings.

Given the high risk of spuriously elevated in-clinic measurements in the severely obese, we examined casual blood pressure measurements performed in large Canadian bariatric program, comparing these measurements to readings performed as part of a prospective clinical trial and taken while closely adhering to contemporary measurement recommendations [13]. Our hypothesis was that mean casual clinic measurements would be, at minimum, 5 mmHg higher than measurements performed under standardized conditions.

## 2. Methods

### 2.1. Subjects

Data from the first 184 patients enrolled in the evaluating self-management and educational support in severely obese patients awaiting multidisciplinary bariatric care (EVOLUTION) randomized controlled trial were analysed. These patients were prospectively enrolled in the study between June 2013 and June 2014. This nine-month trial, consisting of three study arms, has been previously described in detail [17]. EVOLUTION is designed to examine the effectiveness of two interventions (group based education and online education) compared to usual care (mailed education) in adult (age ≥ 18 years) patients wait-listed for bariatric care in Alberta, Canada. Outcomes include body weight, obesity-related comorbidity (including blood pressure), humanistic endpoints, and cost-effectiveness. The University of Alberta Research Ethics Board approved the study protocol and informed consent was obtained from all participants.

Patients analyzed herein were recruited from the Edmonton Weight Wise program, which is a population-representative bariatric care program that has a central, region-wide, single-point-of-access referral system and serves a catchment population of 1.5 million. Weight Wise was established in 2005 and delivers integrated, patient-focused, evidence-based, interdisciplinary bariatric care (consisting of medical treatment and, after 6–12 months, bariatric surgery in eligible candidates) to severely obese patients.

### 2.2. Data Elements

Data from the second EVOLUTION study visit were used. This visit took place three months after baseline enrolment and was scheduled to closely correspond to the initial Weight Wise clinic visit. We excluded patients whose EVOLUTION study visit was more than two weeks before or after their baseline clinic visit.

Data collection was performed by a trained research assistant and included sociodemographics, self-reported medical history, anthropometrics, and blood pressure. Body weight was measured to the nearest 0.1 kg using a calibrated electronic bariatric scale (Scale Tronix, White Plains, New York) and height was measured to the nearest 0.1 cm using a wall-mounted stadiometer.

### 2.3. Standardized Blood Pressure Measurements

Blood pressure measurements in EVOLUTION were performed according to recommended guidelines using an oscillometric blood pressure device (Watch BP Office, Widnau, Switzerland) [17]. Three readings were taken simultaneously in both arms after five minutes of rest, with the patient alone in a quiet room. Care was taken to ensure a proper sized cuff using the measurement range imprinted on the blood pressure cuff as the guide. Arm circumference was not explicitly measured. If the arm was larger than the largest Watch BP cuff size (32–52 cm), blood pressure was taken on the forearm (these patients were excluded from the present analysis). The first reading was discarded and the latter two averaged. For this analysis, in accordance with current recommendations, the arm with the higher mean was used for the analysis [13].

### 2.4. Casual In-Clinic Blood Pressure Measurements

In-clinic casual blood pressure readings were abstracted for EVOLUTION patients between September and October 2014 from the Weight Wise clinic. Clinic measurements were taken with a Welch Allyn Vital Signs Monitor 300 Series or Welch Allyn Connex Vital Signs Monitor (Skaneateles Falls, New York) oscillometric blood pressure device. All cuff sizes, including the large adult size 12 (32–43 cm) and the thigh cuff size 13 (40.7–55 cm) were available. Standard practice in the clinic is to take a single reading in either arm with the patient in a seated position (usually performed by a nurse). A five-minute resting period is usually not observed, proper positioning is not routinely emphasized, and providers may or may not leave the room during the measurement. Service records indicated that the clinic devices were all statically calibrated between 2012 and 2014. Neither arm circumference nor information on the arm used for measurement (left versus right) was available. This represents what we believe to be “usual” clinical practice, with the exception that larger cuffs were routinely available.

### 2.5. Subgroup Analysis

A subgroup analysis in the patients who had their in-clinic visit on the same day as the EVOLUTION study visit was performed. This analysis was also stratified by sequence of initial measurement (in-clinic measurement first followed by study visit measurement later that same day or vice versa).

### 2.6. Statistical Analysis

Descriptive analyses were first performed, including calculation of baseline characteristic means, medians, and proportions. Mean casual clinic blood pressure was compared to the mean standardized blood pressure taken in the EVOLUTION trial, with the latter considered the reference standard. Paired *t*-tests were used to compute *p* values. *p* values < 0.05 were considered statistically significant. Bland-Altman plots were generated to compare the casual in-clinic measurements to the standardized trial measurements across the range of blood pressure values [18]. Pearson's correlation coefficients were also calculated for systolic and diastolic blood pressure readings. A chi-square test was used to compare the proportion of patients in each group with elevated BP (defined as BP levels ≥ 140 and/or ≥90 mmHg).

## 3. Results

### 3.1. Exclusions and Final Sample Size

Of the initial 184 patients, 7 (3.8%) were excluded because they did not have a blood pressure measurement recorded in their clinic chart. Additional 37 (20%) patients were excluded because their arm size was too large to be measured with the Watch BP Office. Of the remaining 140 patients, six patients were excluded because the clinic and EVOLUTION trial visit separated by more than two weeks. The remaining 134 patients were included in the analysis. Of these, 124 (93%) had their initial assessment at the Weight Wise clinic on the same day as the EVOLUTION trial visit. Sixty-seven of 124 patients (54%) had their in-clinic visit (and blood pressure measurement) prior to their study visit and the order was reversed in the remaining patients.

### 3.2. Baseline Characteristics

Baseline characteristics are summarized in Table 1. Mean age was 41.5 ± 8.9 years, mean BMI was 46.8 ± 6.5 kg/m^2^, 101 (75%) were females, and 40 (30%) had a prior history of hypertension.

### 3.3. Blood Pressure Comparisons

Mean blood pressure is summarized in Table 2. Mean casual clinic blood pressure was 128.8 ± 14.1/81.6 ± 9.9 mmHg. Mean standardized study blood pressure was 133.2 ± 15.0/82.0 ± 10.3 mmHg (difference of −4.3 ± 12.0 for systolic (*p* < 0.0001) and −0.4 ± 10.0 mm Hg for diastolic BP (*p* = 0.6)). BP differences (casual minus standardized) were similar in males (−5.3 ± 10.8/−0.7 ± 9.3 mmHg) and females (−4.0 ± 12.4/−0.3 ± 10.3 mmHg) and there was little correlation between the BP differences and BMI (−0.07 for systolic (*p* = 0.4) and (−0.12 for diastolic BP (*p* = 0.2))). The interarm difference in standardized blood pressure was minimal (0.5 ± 10.1/1.1 ± 7.3).

**Figure 1:**
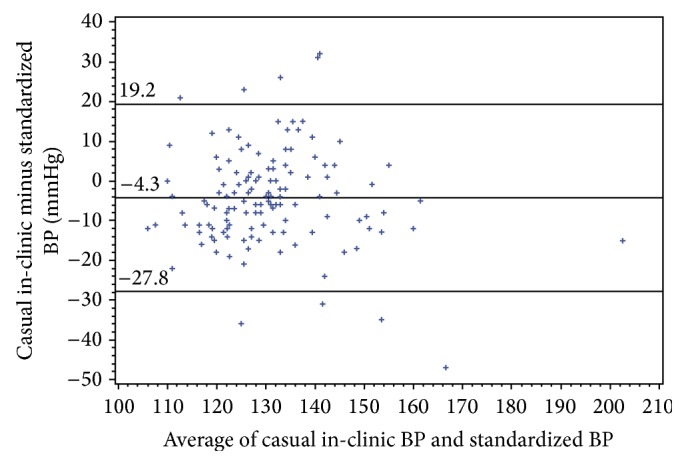
Bland-Altman plot assessing agreement for systolic blood pressure.

**Figure 2:**
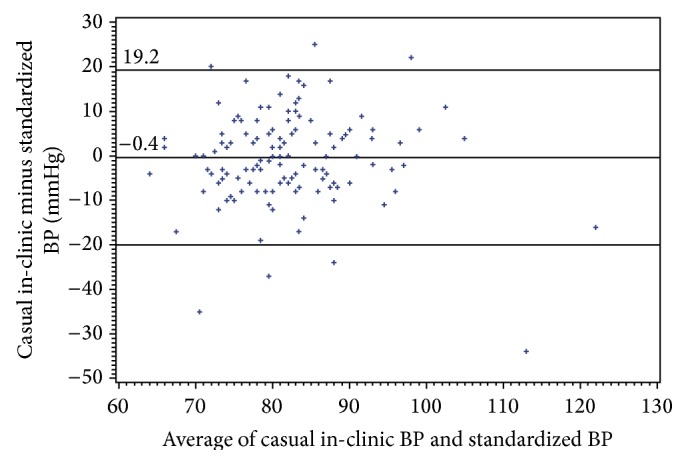
Bland-Altman plot assessing agreement for diastolic blood pressure.

Bland-Altman plots for systolic and diastolic BP showed considerable variability (Figures 1 and 2). As systolic blood pressure levels increased above ≈144 mmHg, the casual in-clinic measurements were consistently lower than the standardized trial measurements (Figure 1), but this was based on a limited number of data points. Systolic readings were strongly correlated (Pearson's correlation 0.66; *p* < 0.0001) and diastolic readings were moderately correlated (0.50; *p* < 0.0001).

Results were similar in the subgroup of 124 patients who underwent same-day in-clinic and trial visit assessments (systolic BP difference of −4.7 ± 12.3 (*p* < 0.001) and diastolic BP difference of −0.6 ± 10.2 (*p* = 0.5)). In the 67 patients who had in-clinic measurement performed first on that day, mean systolic blood pressure differences (in-clinic minus standardized trial BP) were −3.7 ± 10.1 mmHg (*p* = 0.002) and mean diastolic differences were 0.7 ± 8.2 (*p* = 0.4) mmHg. Corresponding numbers were −5.8 ± 14.3 (*p* = 0.004) for systolic blood pressure and −0.4 ± 12.2 (*p* = 0.5) for diastolic blood pressure in the remaining 57 patients who had same-day BP assessments but in whom in-clinic measurement was performed second.

### 3.4. Potential Differences in Treatment Thresholds

In terms of potential treatment thresholds and clinical decision-making, 38 (28.4%) of patients in our cohort had a blood pressure greater than 140/90 mmHg when evaluated by casual in-clinic measurement compared with 36 (26.9%) of those when evaluated using the standardized approach that we considered the “reference” standard (*p* < 0.0001).

## 4. Discussion

In summary, casual blood pressure oscillometric measurements taken in a large bariatric clinic were compared to guideline-concordant standardized oscillometric measurements performed within a prospective randomized trial in 134 severely obese patients with a mean BMI of 47 kg/m^2^. Mean casual in-clinic systolic readings were 4.3 mmHg lower than mean standardized trial measurements and this underestimation relative to standardized readings was greater as blood pressure increased. Diastolic blood pressure readings were comparable between the two types of measurements. Part of the underestimation was attributable to an ordering effect, as the underestimation was 1.7 mmHg greater in patients with same-day measurements in whom clinic measurement was performed last. However, this ordering effect does not explain the overall results. The systolic blood pressure results were unexpected and contrary to our original hypothesis. The other major finding was that there was considerable variability between the two types of blood pressure measurements, with wide limits of agreement for both systolic and diastolic blood pressure. Nevertheless, Pearson's correlations between casual and standardized measurements were high. Furthermore, both casual and in-clinic measurements resulted in similar proportions of patients with elevated BP levels.

We are not aware of any previously published studies that have compared casual in-clinic “real world” blood pressure measurements to standardized measurements in severely obese patients. Studies in nonseverely obese samples have reported contrary findings to the present study; that is, casual measurements were consistently higher than standardized measurements [19, 20]. Higher readings would be consistent with the expected direction of bias, given that most of the aforementioned reasons for inaccurate blood pressure measurement result in falsely elevated readings. One would expect an even greater amount of potential bias in the severely obese because of the known issues related to arm circumference, length, and shape that can predispose to spuriously elevated readings and because only a single clinic reading was taken compared to three standardized measurements (with the first discarded and the latter two averaged) [10–12, 21].

What are the potential explanations for our findings? We postulate that clinic providers, knowing that the arm circumference is likely to be high, may be using the extra large (thigh sized) BP cuff routinely in most patients. Arm circumference is not routinely measured in the clinic and we are also unsure to what extent the clinic providers are using the arm circumference guide imprinted on the cuff. Thus, the possibility of systematic overcuffing, rather than the undercuffing commonly found in nonobese patients, is raised. This may be specific to bariatric clinics as opposed to other primary care or speciality clinics, as bariatric clinics are probably more likely to have and use extra large sized equipment. Another possibility is that the standardized trial visit induced a greater “white coat” response than the in-clinic visit, although this seems unlikely given that the patients are more often anxious in the clinic environment (where their weight is measured and analyzed in detail) and because they were left alone in a quiet room when the standardized trial measurements were taken. A third possibility is that, in this severely obese study population with very high mean BMI levels, the Watch BP Office device readings are high. We note that the extra large (32–52 cm) cuff has met validation criteria, passing all three phases of the international protocol of the European Society of Hypertension [22]. However, the mean arm circumference in this validation study was near the lower limit of the range (36 ± 5 cm) and BMI in this study was relatively low in comparison to our study sample (34 kg/m^2^, with a mean weight of 100.9 kg and height of 1.72 m; Dr. Paolo Palatini, personal communication). Thus, accuracy in the severely obese is not guaranteed and further validation of this (and other) device specifically in this population is necessary.

The present study has several limitations. First, we did not measure arm circumference. Second, in-clinic measurements were collected as part of routine clinical care and not according to a study protocol. In addition, although the in-clinic measurements were almost certainly not performed in standardized fashion (based upon our discussions with clinic staff and direct observation in this and other local settings), these measurements were not observed and, as such, we could not systematically catalogue the protocol deviations (e.g., cuff sizing, patient position, talking during readings, and rest time prior to measurement) that may have occurred. Third, a comparison of two different oscillometric devices was performed; differences in proprietary algorithms may cause differences in blood pressure readings between these two devices [23]. Finally, 20% of the study sample was excluded because a standardized measurement could not be performed in their upper arm; thus, our results may not be generalizable to the heaviest of bariatric patients.

In summary, we unexpectedly found that casual in-clinic blood pressure measurements were, on average, lower than standardized measurements in severely obese patients. This raises the possibility of overcuffing as a potential explanation for our findings (provided our assumption that the standardized trial measurements are an accurate representation of the reference standard). Based upon these findings, an increased risk of overdiagnosis and overtreatment of hypertension in patients seen in this bariatric clinic was not found. However, we do recommend that providers caring for severely obese patients be vigilant about potential “default overcuffing” and take care to ensure that the proper sized cuff is used for blood pressure measurement.

## Figures and Tables

**Table 1:** Baseline characteristics.

Variable	Result
Age (years), mean ± SD	41.5 ± 8.9
Female, number (%)	101 (75)
Weight (kg), mean ± SD	132.5 ± 25.0
BMI (kg/m^2^), mean ± SD	46.8 ± 6.5
Diabetes, number (%)	26 (19)
Dyslipidemia, number (%)	24 (18)
Hypertension, number (%)	40 (30)
Sleep Apnea, number (%)	44 (33)
Smoker (past or current), number (%)	51 (38%)

**Table 2:** Blood pressure results.

Measurement	Mean casual in-clinic BP (mmHg)	Mean standardized trial BP (mmHg)	Mean difference (mmHg)	*p* value
Systolic BP	128.8 ± 14.1	133.2 ± 15.0	−4.3 ± 12.0	<0.0001
Diastolic BP	81.6 ± 9.9	82.0 ± 10.3	−0.4 ± 10.0	0.6
